# 919. Rates of False-Positive Hepatitis B Surface Antigen Is Low in Cancer Patients

**DOI:** 10.1093/ofid/ofab466.1114

**Published:** 2021-12-04

**Authors:** Joseph Klingen, Marcel Yibirin, Jessica P Hwang, Harrys A Torres

**Affiliations:** 1 M.D. Anderson, Houston, Texas; 2 : Departments of Infectious Diseases, Infection Control and Employee Health, houston, Texas; 3 The University of Texas MD Anderson Cancer Center, Houston, TX

## Abstract

**Background:**

Accurate interpretation of hepatitis B virus (HBV) laboratory testing is paramount in avoiding inaccurate diagnosis and incorrect management that could lead to unnecessary and overtreatment. This is particularly relevant in patients with cancer where universal testing is recommended in order to avoid HBV reactivation. Hepatitis B surface antigen testing (HBsAg) positivity indicates chronic or acute HBV infection. The rates and outcomes of a false-positive HBsAg have not been established for patients with cancer.

**Methods:**

Three hundred and ninety-seven patients with any type of cancer and positive HBsAg seen at MD Anderson Cancer Center from January 2016 – January 2021 were retrospectively reviewed in this study approved by the institutional review board. Cases of false-positive HBsAg were defined as those patients with a positive HBsAg but negative HBsAg quantitative, negative HBV core antibody (total Ig), and undetectable HBV DNA within 30 days of positive HBsAg testing. Serum samples from patients were tested for HBsAg using Vitros Enhanced Chemiluminescent Immunoassay (Ortho-Clinical Diagnostics, Raritan, NJ, USA). Data collection includes demographics, past medical history, underlying cancer and its stage, prior cancer treatment, risk factors for HBV, co-infections (hepatitis C, HIV), symptoms, liver function tests, anti-HBV treatment, and interruptions on cancer treatment.

**Results:**

Out of 397 patients with a positive HBsAg, 33 were excluded as they did not meet the diagnostic criteria or have insufficient HBV data. Of them, 3 cases (0.8%) were identified as false positive HBsAg. All 3 patients were female, white, and had progressive malignancy (Table 1). No prior history of liver disease or liver function abnormalities were noted with these 3 patients. Initially, antiviral treatment was started on 1 patient which was discontinued shortly after confirmation of false-positive HBsAg. All 3 patients had additional workup and evaluation by an HBV specialist. In 2 patients, cancer treatment was canceled or delayed.

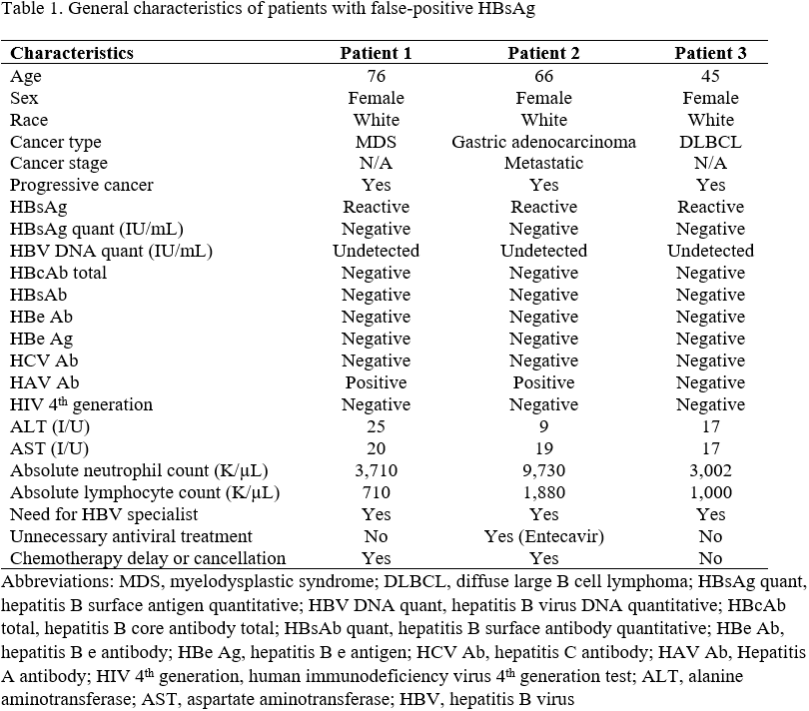

**Conclusion:**

Although uncommon, cancer patients with false-positive HBsAg need further workup to avoid overtreatment and unnecessary interruptions in cancer care

**Disclosures:**

**Jessica P. Hwang, MD, MPH** , **Merck** (Grant/Research Support)

